# Potential preventive effects of forest bathing/*Shinrin-yoku* on depressive symptoms: a narrative review of mechanistic evidence

**DOI:** 10.1265/ehpm.26-00028

**Published:** 2026-07-10

**Authors:** Qing Li

**Affiliations:** Department of Rehabilitation Medicine, Graduate School of Medicine, Nippon Medical School, Tokyo, Japan

**Keywords:** Depression, Depressive symptoms, Forest bathing, Mental health, Immune function, Serotonin, Shinrin-yoku, Sleep, Stress

## Abstract

Depression affects more than 280 million people worldwide and remains a major public health challenge, with limited universally effective preventive strategies. Non-pharmacological interventions have gained increasing attention as complements or alternatives to pharmacotherapy. *Shinrin-yoku* (forest bathing), a Japanese health practice involving immersion in forest environments, has been reported to relieve stress, enhance immune function, and improve mood. This review synthesizes current evidence on the potential preventive effects of forest bathing on depressive symptoms and examines the mechanistic pathways underlying these effects. A structured search of major scientific databases identified studies investigating psychological responses, neuroendocrine and autonomic regulation, immune-inflammatory markers, and related physiological parameters. Across diverse populations, forest bathing consistently reduced negative mood states, improved vigor, and showed beneficial effects on anxiety, depressive symptoms, sleep quality, and stress biomarkers. Mechanistic evidence suggests that forest bathing may modulate the psycho-neuro-endocrino-immune network through improved sleep, reduced stress hormone levels, enhanced parasympathetic activation, and increases in circulating serotonin, oxytocin, and insulin-like growth factor-1, alongside reduced inflammatory markers. While existing findings are promising, most studies involve small samples, short-term interventions, and limited clinical populations. Future large-scale, controlled studies are needed to confirm long-term effects and to clarify whether forest bathing can contribute to prevention or adjunctive management of depressive symptoms.

## Graphical Abstract

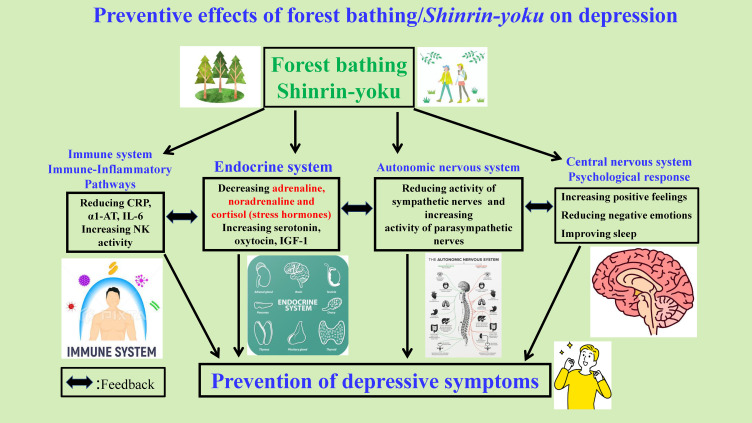

## 1. Introduction

Mental disorders such as psychological stress, anxiety, and depression are widely recognized as public health concerns and are leading causes of disability worldwide [1]. In 2019, an estimated 280 million people (about 5% of adults) experienced depression. Depression is more common in women than men. Globally, more than 10% of pregnant and postpartum women experience depression [2]. Depression prevention has thus become a major social and medical issue worldwide, and new effective methods for preventing depression are needed. Non-pharmacological interventions have become central to contemporary mental health care, serving as complements or alternatives to pharmacotherapy, particularly for mild-to-moderate depression and anxiety [3]. *Shinrin-yoku* translated as ‘forest bathing’, originated in Japan in 1982 and is gaining attention as a way to relieve stress, promote mental health, and prevent disease [4–7]. Forest bathing is a short, leisurely visit to a forest to relieve stress and improve health, and has similar effects to natural aromatherapy, involving the inhalation of volatile substances called phytoncides (aromas) derived from trees [4–12].

Forest bathing has been reported to enhance anti-cancer immune function, suppress sympathetic nervous activity, activate the parasympathetic nervous system, reduce mental stress, stress hormones, and negative moods, increase positive moods, and improve depressive symptoms in both men and women [4–21]. Forest bathing as a non-pharmacological intervention may therefore be useful for improving depressive symptoms and related stress physiology. It has been reported that both male and female patients with depression have low blood serotonin levels [22–27]. Our recent studies indicated that forest bathing significantly increased serum serotonin levels and significantly improved depressive symptoms and sleep quality in healthy males [28] and females with depression [29].

These findings strongly suggest that forest bathing has the potential to improve depressive status. Based on the above background, recently we conducted a systematic review and meta-analysis of randomized controlled trials based on the PRISMA (Preferred Reporting Items for Systematic Reviews and Meta-Analyses) and confirmed the effectiveness of forest bathing in improving mood [30]. There are also several reviews on the effect of forest bathing on mental health [31, 32]. However, there has been no study on the effect of forest bathing on depressive status from the mechanism of forest bathing on human physical and mental health. This review examines the potential preventive effects of forest bathing on depression and the potential underlying mechanisms from the effects of forest bathing on the psycho-neuro-endocrino-immune network [6]. This review is not a systematic review, but a narrative review and focuses on the mechanisms underlying the depressive symptoms-preventing effects of forest bathing.

In addition, this review aims to highlight the importance of forest bathing in determining new directions for non-pharmacological prevention or treatment of mild depression (depressive states). If the preventive effect of forest bathing on depression is confirmed, it is hoped that the importance of promoting and applying forest bathing will be widely understood.

## 2. Search strategy and review methodology

The concept of forest bathing was first proposed in Japan in 1982 [4–8], and research into its stress-relieving, health-promoting, and disease-preventing effects began in the 1990s [6]. Electronic databases (PubMed, Scopus, and Web of Science) were searched (1990–2026) using combinations of terms related to “shinrin-yoku” OR “forest therapy” OR “forest bathing” AND “depression” OR “anxiety” OR “POMS”.

**Inclusion/exclusion criteria**

**Fig. 1:**
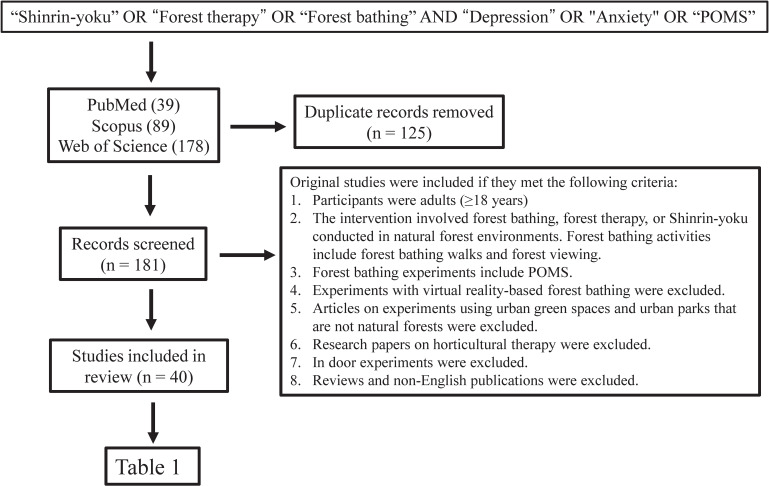
Flowchart of articles screening and inclusion/exclusion criteria

As shown in Fig. 1, original studies were included if they met the following criteria:

1. Participants were adults (≥18 years)
2. The intervention involved forest bathing, forest therapy, or Shinrin-yoku conducted in natural forest environments. Forest bathing activities include forest bathing walks and forest viewing.
3. Forest bathing experiments include POMS.
4. Experiments with virtual reality-based forest bathing were excluded.
5. Articles on experiments using urban green spaces and urban parks that are not natural forests were excluded.
6. Research papers on horticultural therapy were excluded.
7. In door experiments were excluded.
8. Reviews and non-English publications were excluded.

For the mechanism of forest bathing on depressive status, ‘stress hormones’, ‘adrenaline’, ‘noradrenaline’, ‘cortisol’, ‘serotonin’, ‘oxytocin’, ‘sleep’, ‘autonomic nervous system’, ‘sympathetic nervous’, ‘parasympathetic nervous’, ‘immune function’, and ‘natural killer (NK) activity’ were also used to collect the effects of forest bathing on these factors.

## 3. Effect of forest bathing on the symptoms of depressive status

Research into the preventive effects of forest bathing on depression has generally been conducted using the Profile of Mood States (POMS) test developed by McNair et al. [33]. The POMS is a 65-item self-administered questionnaire designed to assess six mood states: tension-anxiety, anger-hostility, fatigue, depression, vitality, and confusion. Yokoyama et al. [34] developed a Japanese version and reported acceptable levels of reliability and validity. Recently, a shortened version of the POMS with 35 items has been widely used [28–30]. The POMS test has been used to evaluate the effects of forest bathing on depressive symptoms both in depressive patients or subjects with depressive tendencies [29, 35, 36] and in healthy subjects [8, 10–21, 28, 37–63].

### 3-1 Effects of forest bathing time on POMS scores

As shown in Table 1, previous studies have reported the benefits of forest bathing for 15–20 minutes [17–21, 37–40], 30 minutes [40–42], 1 hour [43], 90 minutes [44, 45], 2 hours [36, 46–50], 3 hours [51], 1 day [11, 15, 16, 28, 29, 35, 52, 54], 2 days [53], 3 days [8, 10, 56–61], 4 days [62] and 7 days [63] on POMS scores. Positive effects of forest bathing were observed in all studies, even though the subjects and the types of forest differed. In summary, forest bathing significantly increased the score for vigor and decreased the scores for anxiety, depression, anger, fatigue and confusion in the POMS test and produced relaxation effects in both males and females. The anti-anxiety, antidepressant, and relaxation effects of forest bathing, as measured by the POMS test, begin to appear 15–20 minutes after forest bathing and reach a peak at two hours. Two hours of forest bathing is sufficient for the relaxation effects. Females showed more of an effect than males [6, 28, 29].

**Table 1:** The main references on the effects of forest bathing on depression determined by POMS

**Authors**	**Journals**	**Methods and designs**	**Subjects (number and age)**	**Observation period (duration)**	**Main outcomes (POMS)**	**Ref. No.**
Park et al.,	Environ Health Prev Med 2010;15:18–26.	Forest vs city by randomized crossover design#	N = 280, aged 21.7 ± 1.5, male university students	20 minutes	Decreases of negative emotions and increase of positive feeling in POMS	17
Lee et al.	Public Health. 2011;125(2):93–100.	Forest vs city by randomized crossover design	N = 12 (males, 21.2 ± 0.9)	15 minutes	Decreases of negative emotions and increase of positive feeling in POMS	18
Song et al	Int J Environ Res Public Health. 2018;15(12):2804.	Forest vs city by randomized crossover design	N = 585, aged 21.7 ± 1.6 years, males	15 minutes	Decreases of negative emotions and increase of positive feeling in POMS Decrease of anxiety in State−Trait Anxiety Inventory	19
Song et al	Int J Environ Res Public Health. 2019;16(2):229.	Forest vs city by randomized crossover design	N = 60, aged 21.0 ± 1.3, females	15 minutes	Decreases of negative emotions and increase of positive feeling in POMS Decrease of anxiety in State−Trait Anxiety Inventory	20
Song et al	J. Environ. Res. Public Health 2015;12(3):2687–2699.	Forest vs city by randomized crossover design	N = 20, aged 58.0 ± 10.6, males	17 minutes	Decreases of negative emotions and increase of positive feeling in POMS	21
Joung et al.	Int J Environ Res Public Health. 2020;17(9):3266.	Forest vs city by randomized crossover design	N = 22 (15 males and 9 females, aged 20.9 ± 1.3)	15 minutes	Decreases of negative emotions and increase of positive feeling in POMS Decrease of anxiety in State−Trait Anxiety Inventory	37
Bielinis et al.	PLoS One. 2021;16(1):e0244799.	Forest vs non-forest by randomized crossover design	N = 22 (11 females, 11 males, aged 22.5 ± 4.67)	5 minutes walking+ 15 minutes viewing	Decreases of negative emotions and increase of positive feeling in POMS	38
Ramanpong et al.	TREES FORESTS AND PEOPLE, 2014, 16, 10.1016/j.tfp.2024.100551	Viewing forests vs city by randomized controlled trial without crossover	N = 74 (females:45, males: 29) Age: 20–65	15 minutes	Decreases of negative emotions and increase of positive feeling in POMS	39
Sun et al.	Front Public Health. 2024;12:1296714.	Forest viewing (before vs after)	N = 90, aged 18.57 ± 0.94, First-year students in university	20 and 30 minutes	Decreases of Total Mood Disturbance (TMD) in POMS	40
Takayama et al.	Int J Environ Res Public Health. 2014;11(7):7207–30.	Forest vs city by randomized crossover design	N = 45, aged 21.1 ± 1.3, male university students	30 minutes (15 min walking, 15 min viewing)	Decreases of negative emotions and increase of positive feeling in POMS	41
Takayama et al	Int J Environ Res Public Health. 2019;16(8):1456.	Forest vs city by randomized crossover design	N = 46, Males, 21.4 ± 1.3	30 minutes (15 min walking, 15 min viewing)	Decreases of negative emotions in POMS	42
Oomen-Welke et al	Int J Environ Res Public Health. 2022;19(22):15322.	Forest bathing (before vs after)	N = 39, aged 18–70, 44.7 ± 15.8, females: 35, males: 4	1 hour	Decrease of depression in POMS	43
Bressane et al.	Int J Environ Res Public Health. 2025;22(4):579.	Forest bathing (before vs after)	N = 89 (18–60)	90 minutes	Decrease of depression and anxiety in POMS	44
Ochiai et al.	Sci Rep. 2025;15(1):3272.	Forest vs city by randomized controlled trial without crossover	n = 37, forest bathing n = 41, city walking men aged 40–70	90 minutes	Decreases of negative emotions and increase of positive feeling in POMS	45
Furuyashiki et al	Environ Health Prev Med. 2019;24(1):46.	Forest bathing (before vs after)	N = 155, 37% with depressive tendencies (n = 58, 44.0 ± 9.6, 19–59)	2-hour	Decreases of negative emotions in POMS Depressive tendencies greater improvement in POMS than those without depressive tendencies.	36
Ochiai et al.	Int J Environ Res Public Health. 2015;12(3):2532–42.	Forest bathing (before vs after)	N = 9, male, aged 56 ± 13.0	2-hour	Decreases of negative emotions in POMS	46
Ochiai et al.	Int J Environ Res Public Health. 2015;12(12):15222–32.	Forest bathing (before vs after)	N = 17, aged 62.2 ± 9.4, females	2-hour	Decreases of negative emotions in POMS	47
Fu et al.	Int J Environ Res Public Health. 2022;19(3):1231.	Forest bathing (before vs after)	N = 38 (24 females, 14 males, aged 43.55 ± 11.61, healthy participants)	2-hour	Decreases of negative emotions and increase of positive feeling in POMS	48
Yu et al.	Int J Environ Res Public Health. 2017;14(8):897.	Forest bathing (before vs after)	N = 128, aged 60.0 ± 7.44, males = 43, females = 85	2-hour	Decreases of negative emotions, increase of vigor-activity in POMS	49
Wei et al.	Environ Health Prev Med. 2026, 31, 7	Forest bathing (before vs after)	N = 38, aged 61.0 (52.0–65.0), males = 11, females = 27	2-hour	Decreases of negative emotions, increase of vigor-activity in POMS	50
Serrat et al.	Healthcare (Basel) 2025;13(14):1654.	Forest bathing (before vs after)	N = 44, aged 53.82 ± 8.57, females	3-hour	Decreases of negative emotions, increase of vigor-activity in POMS	51
Li et al	J Biol Regul Homeost Agents 2010;24:157–165.	Forest bathing (before vs after)	N = 12, healthy male subjects, aged 35–53 years, 44.4 ± 5.7	One-day	Decreases of negative emotions and increase of positive feeling in POMS	11
Li et al	Eur J Appl Physiol. 2011;111(11):2845–53.	Forest vs city by randomized controlled trial without crossover	N = 16, healthy male subjects, aged 57.4 ± 11.6 years	One-day	Decreases of negative emotions and increase of positive feeling in POMS	15
Li et al	Evid Based Complement Alternat Med. 2016;2016:2587381	Forest vs city by randomized controlled trial without crossover	N = 19, male subjects, aged 40–69 years, 51.2 ± 8.8	One-day	Decreases of negative emotions and increase of positive feeling in POMS	16
Li et al.	Environ Health Prev Med. 2022;27:44.	Forest vs city with randomized crossover design	N = 20, males, aged 57.3 ± 8.4 years	One-day	Decreases of negative emotions and increase of positive feeling in POMS	28
Li et al.	Diseases. 2025;13(4):100.	Forest vs city with randomized crossover design	N = 31, 40.1 ± 2.4 years Females with depression	One-day	Decreases of negative emotions and increase of positive feeling in POMS	29
Bielinis et al.	Int J Environ Res Public Health. 2019;17(1):118.	Forest bathing (before vs after)	N = 50, aged 42.44 ± 13.23 females: 27, males: 23, psychotic or affective disorders	One-day	Decreases of negative emotions and increase of positive feeling in POMS	35
Mao et al.	Biomed Environ Sci. 2012;25(3):317–24.	Forest (N = 10) vs city (N = 10) by randomized controlled trial without crossover	N = 20, aged 20.79 ± 0.54, male university students	One-day	Decreases of negative emotions and increase of positive feeling in POMS	52
Li et al.	J Occup Health. 2025;67(1):uiaf041.	Forest vs city by randomized crossover design	N = 30, aged 63.1 ± 7.5, males	One-day	Decreases of negative emotions and increase of positive feeling in POMS	54
Chen et al.	FORESTS 2018, 9(7), 403	Forest bathing (before vs after)	N = 16, aged 46.88 ± 7.83 years, females	2-day/1-night	Decreases of negative emotions and increase of positive feeling in POMS	53
Li et al	Int J Immunopathol Pharmacol. 2007;21:117–27.	Forest bathing (before vs after)	N = 12, healthy male subjects, aged 35–56 years	3-day/2-night	Decreases of negative emotions and increase of positive feeling in POMS	8
Li et al	J Biol Regul Homeost Agents. 2008;22:45–55.	Forest bathing (before vs after)	N = 13, healthy female nurses, aged 25–43 years	3-day/2-night	Decreases of negative emotions and increase of positive feeling in POMS	10
Li et al.	Front Public Health. 2025;13:1631613.	Forest vs city by randomized controlled trial without crossover	Forest (N = 24: M/F = 13/11, 71.92 ± 6.06), city (N = 12: M/F = 7/5, 73.75 ± 6.41)	3-day/2-night	Decreases of negative emotions and increase of positive feeling in POMS	56
Weng et al.	Forests 2024, 15(2), 393	Forest bathing (before and after)	N = 41, aged 32.17 ± 8.08 (males = 21, female = 20)	3-day/2-night	Decreases of negative emotions and increase of positive feeling in POMS	57
Quan et al.	FRONTIERS IN PUBLIC HEALTH 2024,12 fpubh.2024.1366339	Forest bathing (before vs after)	N = 12, males = 5, females = 7, aged 35–39	3-day/2-night	Decreases of negative emotions and increase of positive feeling in POMS	58
Lyu et al.	Int J Environ Res Public Health. 2019;16(24):4991.	Forest (N = 45) vs city (N = 15) by randomized controlled trial without crossover	Male College Students Aged 19–24	3-day/2-night	Decreases of negative emotions and increase of positive feeling in POMS	59
Dai et al.	Front. For. Glob. Change, 2025 8:1619569.	Forest bathing (before vs after)	N = 36 aged 18–45	3-day/2-night	Total POMS scores improved	60
Jia et al.	Biomed Environ Sci. 2016;29(3):212–8.	Forest vs city with randomized controlled trial without crossover	N = 10 (forest), N = 8 (city) (aged 70 ± 7)	3-day/2-night	Decreases of negative emotions in POMS	61
Mao et al.	Int J Environ Res Public Health. 2017;14(4):368.	Forest vs city by randomized controlled trial without crossover	Forest: N = 23, M/F = 12/11, 72.86 ± 5.85, city: N = 10, M/F = 7/3, 70.70 ± 3.68	4-day	Decreases of negative emotions in POMS	62
Mao et al.	J Cardiol. 2012;60(6):495–502.	Forest (N = 12) vs city (N = 12) by randomized controlled trial without crossover	N = 24, aged 60–75 year	7-day/7-night	Decreases of negative emotions in POMS, not vigor	63

# A randomized crossover design is a research method in which participants receive two or more treatments (interventions) in a random order, reducing the impact of individual differences by having the participants themselves act as a control. In this research design, all participants experience 2 interventions (forest bathing and city walking) sequentially in a randomly assigned order, such as intervention A (e.g. forest bathing) then B (e.g. city walking), or intervention B then A [17–21, 28, 29, 37, 54].

### 3-2 Effects of different experimental research designs for forest bathing on POMS scores

As shown in Table 1, three different research designs have been reported so far: comparisons of POMS scores before and after forest bathing [8, 10, 11, 35, 36, 40, 43, 44, 46–51, 53, 57, 58, 60], comparisons of forest bathing with urban environments using randomized controlled trial without crossover design [15, 16, 39, 45, 56, 59, 61–63], and comparisons of forest bathing with urban environments using a randomized crossover design [17–21, 28, 29, 37, 38, 41, 42, 54]. All designs demonstrated relaxation effects of forest bathing determined by the POMS test.

### 3-3 Effects of forest bathing on different subjects

While most studies have examined the effects of forest bathing on POMS scores in healthy subjects [8, 10, 15–21, 28, 37–63], only three studies have examined the effects of forest bathing on POMS scores in subjects with mild depression or psychiatric disorders [29, 35, 36]. Relaxation effects of forest bathing were confirmed in all subjects. Furuyashiki et al. (2019) [35] investigated the physiological and psychological effects of forest bathing on employed people with and without a tendency toward depression, and found that after forest bathing subjects with a tendency toward depression showed significantly greater improvements in many POMS items than subjects without a tendency toward depression. Bielinis et al. (2019) [36] investigated the effects of forest bathing on patients with affective and psychotic disorders admitted to psychiatric hospitals using the POMS test. In the group of patients with affective disorders, forest bathing had a positive effect on almost all POMS subscales except for the “Anger-Hostility” scale which did not change significantly after the intervention. In these patients, the greatest effects were observed on the “Confusion” and “Depression-Dejection” subscales. In patients with psychotic disorders, the “Confusion” and “Vitality” subscales showed the greatest changes. The observed changes in psychological indicators in psychiatric hospital patients with both types of disorders suggest that forest therapy interventions may have a positive impact on their mental health. Our research team [29] also examined the effect of forest bathing on POMS scores in female participants with depression/depressive tendencies and found that forest bathing significantly reduced the scores for negative moods such as anger–hostility, confusion–bewilderment, depression–dejection, fatigue–inertia, tension–anxiety, and total mood disturbance, and significantly increased positive moods such as vigor–activity and friendliness in the POMS test. Forest bathing also significantly decreased Self-rating Depression Scale (SDS) scores and increased the concentrations of blood serotonin, oxytocin and insulin-like growth factor I (IGF-1). These results suggest that forest bathing is effective in improving depressive symptoms in patients with depression. Despite promising findings, evidence in clinical populations remains limited.

### 3-4 Effects of forest bathing on symptoms of depressive status evaluated by other indicators

Morita et al. [64] investigated the effects of forest trips using the Multiple Mood Scale-Short Form (hostility, depression, boredom, friendliness, wellbeing and liveliness) and found that hostility and depression scores significantly decreased and liveliness scores significantly increased on days of forest bathing compared to control days. They concluded that customary forest bathing may help to decrease the risk of psychosocial stress-related diseases. Other researchers [19, 20, 35, 37, 53] using the State Trait Anxiety Inventory (STAI) questionnaire also confirmed the anti-anxiety effect of forest bathing. SDS [29], blood serotonin [28, 29, 66, 67] and oxytocin [29] were also used to evaluate the effects of forest bathing on depressive status. Kavanaugh et al. [65] used the Oldenburg Burnout Inventory and the Mini-Z questionnaire to assess the impact of *Shinrin-yoku* (forest bathing) on physician/healthcare professional burnout.

Previous systematic review and meta-analysis indicated that forest bathing shows a moderate and clinically meaningful psychological benefit [30]. Considering the potential confounding factors including physical activity (walking component), social interaction during forest trips, removal from urban stressors, expectation/placebo effects, and exposure to sunlight and fresh air, it should claim moderate causal between forest bathing and improvement of depressive symptoms. On the other hand, it also should be understood that the effects of forest bathing are the total effect of the forest environments including the quiet atmosphere, beautiful scenery, calm climate, pleasant aromas, and clean fresh air compared with city environments [4, 7, 29, 54].

## 4. Potential underlying mechanisms of forest bathing on the improvement of depressive symptoms

The above studies consistently demonstrated that forest bathing is effective in improving depressive symptoms. To understand why forest bathing has anti-anxiety, antidepressant, and relaxation effects, we can explore the preventative effects of forest bathing on depressive symptoms through several potential mechanisms.

### 4-1. Improving sleep

Sleep disturbances and insomnia are common and important symptoms in most patients with depression [68, 69]. Sleep disorders can trigger or worsen depression [70, 71]. Forest bathing has positive benefits for sleep [6, 28, 29, 54, 64, 72–76]. We previously reported that forest bathing significantly increased sleep time in middle-aged male subjects [6], and recently found that forest bathing significantly improved sleep quality in healthy males [28, 54] and females with depression [29]. Morita et al. reported that two hours of forest bathing improved nocturnal sleep conditions for individuals with sleep complaints, possibly as a result of exercise and emotional improvement [64]. Furthermore, Kim et al. conducted a six-day forest bathing in postmenopausal women and measured their sleep status using polysomnography and sleep questionnaires before and after forest bathing. They found that forest bathing may be an excellent alternative to non-pharmacological therapy for alleviating insomnia in postmenopausal women [76]. Other studies also found that forest bathing may improve sleep [72–75]. These findings suggest that forest bathing may have potential preventive effects on depressive symptoms by improving sleep.

### 4-2. Reducing stress and stress hormones

**Fig. 2:**
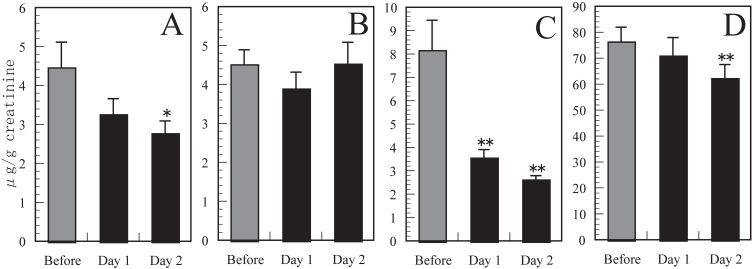
Effect of a forest bathing trip on adrenaline and noradrenaline concentrations in urine. (A): Effect of a forest bathing trip on urinary adrenaline concentrations in males (n = 12). (B): Effect of a city trip on urinary adrenaline concentrations in males (n = 11). (C): Effect of a forest bathing trip on urinary adrenaline concentrations in females (n = 13). (D): Effect of a forest bathing trip on urinary noradrenaline concentrations in females (n = 13). Data are presented as the mean + SE. *: *p* < 0.05, **: *p* < 0.01, significantly different from before the trip by paired *t*-test. Cited from Li et al. Int J Immunopathol Pharmacol. 2008; 21: 117–128 [9] and Li et al. J Biol Regul Homeost Agents. 2008; 22: 45–55 [10] with permission from Biolife.

Stress, especially mental stress, is a strong risk factor for major depressive disorders [77, 78]. Therefore, mental stress and stressful conditions can induce or worsen depression. Of the three stress hormones, adrenaline mainly indicates mental stress while noradrenaline mainly indicates physical stress and cortisol can indicate both mental and physical stress [7, 9–11, 15–18, 37, 45]. As shown in Fig. 2, our research team previously reported that forest bathing decreased stress hormones, and may thereby contribute to stress management [9–11, 15, 16]. Other researchers have also reported that forest bathing decreased saliva cortisol levels [17, 18, 37, 45].

Stress and sleep disorder are two keys to understanding the background of depression and the relationship with forest bathing. Forest bathing can reduce stress and improve sleep [6, 9, 10, 28, 29, 54]. Therefore, forest bathing may have a potential preventive effect on depressive symptoms by reducing mental stress and by improving sleep.

### 4-3. Reducing the activity of sympathetic nerves and increasing parasympathetic activity

Major depressive disorder is associated with an autonomic nervous system imbalance. All the symptoms of depression (high cortisol, high adrenaline, insomnia, agitation, anxiety) can probably be attributed to over-activation of the sympathetic nervous system [79, 80]. Therefore, the autonomic balance is also a key factor in understanding the background of depression and the relationship with forest bathing. A higher level of sympathetic activity and disturbance of the autonomic balance can induce depression and sleep disorder [4]. Forest bathing can decrease sympathetic nerve activity, increase parasympathetic activity and stabilize the autonomic balance [15–21]. Therefore, forest bathing may have a potential preventive effect on depressive symptoms by stabilizing the autonomic balance.

Mental stress, autonomic nervous system imbalance, and sleep disorders all influence each other, and all three can trigger or worsen depression. Forest bathing reduces stress, improves sleep, and balances the autonomic nervous system, thereby contributing to the improvement of depressive symptoms.

### 4-4. Increasing serotonin, oxytocin, and insulin-like growth factor I (IGF-1) in blood

Patients with major depressive disorder show lower levels of serotonin in blood [22–27, 29, 81]. There are significant inverse correlations between levels of blood serotonin and the values calculated for degree of depression on the Beck Depression Inventory and degree of anxiety on the Hamilton A questionnaire scale, respectively, in type-2 diabetic patients with anxiety and depression [27]. Our research team found that forest bathing significantly increased the levels of blood serotonin in healthy males [28] and in female patients with depression/depressive tendencies [29]. Park et al. [66] and Kim et al. [67] also reported that forest therapy significantly increased the levels of blood serotonin in middle-aged women and men in Korea, which supports our findings.

Oxytocin plays an important and central role in human social behavior, social cognition, anxiety, mood, stress regulation, fear learning and extinction. The relationship between blood oxytocin levels and psychiatric disorders (e.g., depression, anxiety, and autism spectrum disorders) has been widely studied [82–86]. These findings indicate an association between depressive symptomatology and oxytocin levels. In addition, insulin-like growth factor I (IGF-1) increases the number of new neurons in the hippocampus, contributing to antidepressant effects [87–91]. Pregnant women with higher serum IGF-1 concentrations during early pregnancy are less likely to develop postpartum depression than those with lower concentrations, suggesting that high serum IGF-1 concentrations during pregnancy may protect against the onset of postpartum depression [91]. Taş Dürmüş et al. reported that a high-intensity training program significantly increased serum IGF-1 levels and significantly reduced symptoms of depression and anxiety [92], suggesting a strong association between depression and IGF-1. Based on the above findings, our research team recently conducted a forest bathing study in a Japanese cypress forest using a randomized crossover design to compare the effects of forest bathing with city walking on blood oxytocin and IGF-1, and found that forest bathing significantly increased the levels of oxytocin and IGF-1 in serum in female patients with depression/depressive tendencies [29]. These findings suggest that forest bathing showing potential preventive effect on depressive symptoms may be associated with the increases of serotonin, oxytocin and IGF-1 in blood. However, there is still little research on the effects of forest bathing on human blood serotonin, oxytocin, and IGF-1. Further large-scale and long-term forest bathing studies on this topic are needed in both healthy individuals and patients with depression.

### 4-5. Affecting the immune-inflammatory pathways

Patients with major depression show lower natural killer (NK) activity [93–96]. It has been reported that forest bathing can increase human NK activity by increasing the numbers of NK cells and intracellular anticancer proteins such as perforin, granzymes and granulysin in human NK cells [8–14, 59]. This research topic has emerged as a central paradigm in the biology of mood disorders. Patients with major depression often exhibit low-grade systemic inflammation. Elevated inflammatory markers such as C-reactive protein (CRP), interleukin-6 (IL-6), and tumor necrosis factor-α (TNF-α) are reported to be associated with depression severity, cognitive impairment, and poor treatment response [94]. Depressed patients exhibit alterations in plasma cytokine concentrations, the number and activation levels of immune cells, including higher levels of proinflammatory cytokines, a predominance of Th2 responses over Th1 responses, and weakened NK activity [95]. Our research team recently found that forest bathing significantly decreased the concentrations of blood CRP, α1-AT, IL-6, and fibrinogen, significantly increased SpO_2_, and reduced subjective fatigue and respiratory symptoms in male subjects at risk of developing chronic obstructive pulmonary disease [54]. Jia et al. [61] found that forest bathing decreased levels of proinflammatory cytokines including IL-6, IL-8, interferon-γ, IL-1β, and CRP. Mao et al. [62, 97] also reported that forest bathing reduced the level of IL-6. Kim et al. [67] also found that walking in forests reduced CRP levels in healthy adults. Based on the above evidence, forest bathing may have potential preventive effects on depressive symptoms by affecting the immune-inflammatory pathways.

While the reproducibility of the effect of forest bathing on NK activity among the effects of forest bathing on biomarkers of NK activity, CRP, cytokines (IL-6, etc.), serotonin, oxytocin, and IGF-1 has been well confirmed to show consistent replication [8–14, 59, 98, 99]; however, there are currently relatively few studies on the effects of forest bathing on other biomarkers. Further large-scale and long-term forest bathing studies on this topic are needed in both healthy subjects and patients.

Why does the forest bathing affect human health? What kind of factors in the forest environment contribute to beneficial effects on human health? The quiet atmosphere, beautiful scenery, mild climate, special good smell, and fresh, clean air in forests contribute to the effects. It is the total effect from all five senses: senses of sight, smell, hearing, touch and taste stimulated by the forest environment [6, 7, 28, 29]. In fact, sense of smell by breathing in volatile organic substances, called phytoncides from trees, such as α-pinene and limonene that are present in forests but not in urban settings may partially contribute to the effect [98]. It has been reported that phytoncides from the trees boosted the immune function [98, 99], reduced stress hormones and improved emotions [98]. Kim et al [100] reported that phytoncide odor significantly decreased low and high β activity evaluated by electroencephalography that suggest a positive effect on reductions of anxiety, depression, and stress in patients with mild cognitive impairment. Meta-analysis shows that aroma inhalation [101] and aromatherapy [102] improve sleep quality and alleviate stress, depression, anxiety, and fatigue. The above evidence suggests the importance of phytoncides (aromas) in the mechanism of the prevention of depressive symptoms through forest bathing.

Moreover, forest bathing is a non-pharmacological preventive intervention for depressive symptoms that has no side effects, is free, and is effective in improving depressive symptoms.

**Fig. 3:**
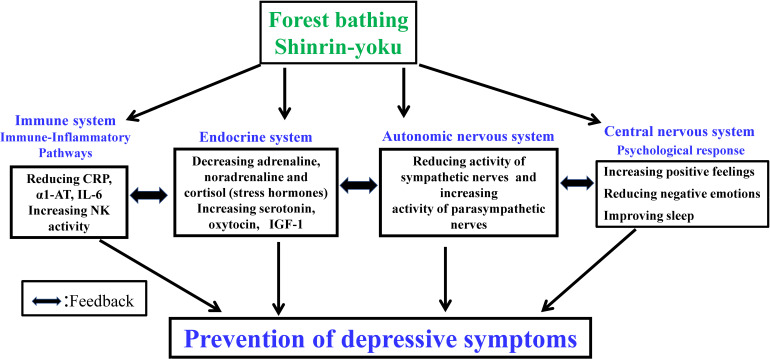
Potential underling mechanism of forest bathing on the prevention of depressive symptoms via the psycho-neuro-endocrino-immune network.

As shown in Fig. 3, forest bathing has potential preventive effects on depressive symptoms by affecting the human immune, endocrine and nervous systems through the psycho-neuro-endocrino-immune network [6]. However, it should be emphasized that evidence supports short-term mood improvement and physiological modulation rather than established prevention of depressive disorder.

## A statement on quality appraisal

In terms of the strength of the evidence, the results of comparative experiments between forest bathing and urban walks using randomized crossover designs are superior to those using randomized controlled trial without crossover design, followed by those comparing forest bathing before and after. In the cited papers in this review, 12 studies (30%) used randomized crossover designs, 10 studies (25%) used randomized controlled trial without crossover designs, and 18 studies (45%) compared forest bathing before and after, indicating a balanced distribution methodologically. However, no formal risk-of-bias assessment was conducted in the present review.

## Limitations

This review has the following limitations:

1. This is a review paper, and because it focuses on the results of various studies on the effects of forest bathing on improving depressive symptoms, methods and sample sizes of studies are not consistent, which may have a risk of bias.
2. Most studies cited involve small sample sizes, short-term exposure (15 minutes to several days), and non-clinical or mildly symptomatic populations. Very few studies involve clinically diagnosed major depressive disorder and long-term follow-up data are absent.
3. In fact, the effects of forest bathing are the total effects of the five senses of sight, hearing, taste, smell and touch, stimulated by the forest environment [4, 7]. However, as the potential confounding factors including physical activity (walking component), social interaction during forest trips, removal from urban stressors, expectation/placebo effects, and exposure to sunlight and fresh air should be considered. This is a limitation of forest bathing research.
4. At present, although forest bathing is recognized as preventive medicine, it is not a clinical methodology. There are no studies comparing the effects of forest bathing (a non-pharmaceutical intervention) on depression with those of pharmacological intervention (antidepressants). Further research is needed in order to discuss and compare these treatment options.

## Conclusions and directions for future research

1. Forest bathing and forest medicine as a non-pharmacological intervention may represent a new strategy, including as a clinical option, for the prevention and treatment of depressive symptoms in relation to the formulation of public health policies.
2. Further controlled and randomized crossover studies and interdisciplinary research are needed in order to explore the effects of forest bathing on depression in patients.
3. Previous research has focused on the effects of short-term forest bathing on depressive symptoms. Further empirical research is needed on the long-term effects of forest bathing with large-scale on depression in patients.
4. Future research is also needed to examine the improvement of depressive symptoms through forest bathing in multiple and international facilities and with large subject groups.
